# Time-resolved proteomic adaptation of multidrug-resistant *Acinetobacter baumannii* to antimicrobial stress induced by partially purified fraction from *Caesalpinia pulcherrima* flower using DEqMS

**DOI:** 10.1128/spectrum.01832-26

**Published:** 2026-07-30

**Authors:** Wanchat Sirisarn, Peerapong Chumkaeo, Thammarit Khamplod, Narumon Phaonakrop, Kiattawee Choowongkomon, Kriskamol Na Jom, Sittiruk Roytrakul, Sompop Saeheng

**Affiliations:** 1 Department of Microbiology, Faculty of Medicine, Kasetsart University 724376, Bangkok, Thailand; 2 NANOCAST Laboratory, Center for Catalysis for Advanced Sustainable Transformation (CAST), Department of Chemistry and Center of Excellence for Innovation in Chemistry, Faculty of Science, Mahidol University, Bangkok, Thailand; 3 Department of Biotechnology, Faculty of Agro-Industry, Kasetsart University 533853, Bangkok, Bangkok, Thailand; 4 National Center for Genetic Engineering and Biotechnology, National Science and Technology Development Agency, Pathum Thani, Thailand; 5 Department of Biochemistry, Faculty of Science, Kasetsart University 54775, Bangkok, Thailand; 6 Department of Food Science and Technology, Faculty of Agro-Industry, Kasetsart University 533853, Bangkok, Thailand; 7 Center of Excellence for Biochemistry, Faculty of Science, Prince of Songkla University, Songkhla, Thailand; University of Technology Sydney, Sydney, Australia

**Keywords:** *Acinetobacter baumannii*, *Caesalpinia pulcherrima*, label-free quantitative proteomics, DEqMS

## Abstract

The global prevalence of multidrug-resistant (MDR) bacteria represents an urgent public health challenge, emphasizing the critical need for novel antimicrobial agents. MDR *Acinetobacter baumannii*, a nosocomial pathogen of critical global concern owing to its capacity to acquire and disseminate antimicrobial resistance, was employed as a bacterial model to investigate the antimicrobial potential of natural products derived from *Caesalpinia pulcherrima* (L.) Sw. (Fabaceae). This medicinal plant represents a promising reservoir of novel bioactive compounds; however, its molecular effects on the *A. baumannii* proteome had not previously been characterized. The partially purified ethyl acetate fraction of *C. pulcherrima* flowers (CPF4) exhibited potent bactericidal activity against susceptible *A. baumannii* (minimum inhibitory concentration and minimum bactericidal concentration = 31.25 µg/mL), and time-resolved label-free quantitative LC-MS/MS proteomics was subsequently performed on MDR *A. baumannii* cultures treated with CPF4 at 24 h, 48 h, and 72 h post-treatment alongside untreated controls in biological triplicate, with differential protein expression assessed using differential expression of quantified mass spectrometry data. No significantly differentially expressed proteins were detected at 24 h or 48 h relative to the control, indicating that the proteomic effects of CPF4 manifest predominantly at the late treatment stage. In contrast, a robust late-phase response was identified at 72 h, comprising the coordinated induction of proteins associated with DNA damage repair, transcriptional regulation, and cell surface glycosylation remodeling. The sensor histidine kinase PmrB was significantly upregulated at 72 h vs 48 h (adjusted *P* = 0.029), implicating the PmrA/PmrB two-component system in late-phase colistin tolerance acquisition under sustained CPF4 exposure.

**IMPORTANCE:**

These findings provide mechanistic insight into the adaptive survival strategies employed by multidrug-resistant *Acinetobacter baumannii* in response to plant-derived antimicrobial challenge and support the further development of *Caesalpinia pulcherrima*-derived natural products as candidate antimicrobial agents.

## INTRODUCTION

*Acinetobacter baumannii* is one of the most clinically problematic nosocomial pathogens globally, classified by the World Health Organization (WHO) as a critical-priority pathogen for which new therapeutic options are urgently required (1). The organism’s clinical significance derives from its extraordinary capacity to acquire and disseminate antibiotic resistance determinants, particularly carbapenem-hydrolyzing beta-lactamases (OXA-type), efflux pump overexpression, porin loss, and target modifications conferring resistance to virtually all available antibiotic classes (2). The emergence of multidrug-resistant (MDR) strains has rendered treatment of *A. baumannii* infections increasingly difficult, with high mortality rates from bloodstream infections in intensive care settings (3). WHO stated that *A. baumannii* is recognized as one of the major nosocomial ESKAPE pathogens due to its remarkable capacity to acquire multidrug resistance, particularly against carbapenems, colistin, and other last-line antibiotics (1, 4). The urgent need for natural anti-*A*. *baumannii* agents, together with their underlying mechanisms of action, remain poorly understood. Proteomic analysis provides a powerful approach to elucidate bacterial responses to antimicrobial treatment by identifying alterations in protein expression and affected cellular pathways, thereby facilitating the discovery of potential therapeutic targets.

Plant-derived natural products have attracted renewed research interest as sources of novel antimicrobial agents and as potential adjuvants capable of sensitizing resistant bacterial isolates to conventional antibiotics (5). *Caesalpinia pulcherrima* (L.) Sw. (Fabaceae), commonly known as the peacock flower, is a pantropical ornamental and medicinal plant with documented broad-spectrum antimicrobial, anti-inflammatory, and antioxidant activities attributable to its rich phytochemical content, including polyphenols, flavonoids, terpenes, and alkaloids (6, 7). While our previous work has reported antimicrobial activities using the whole *C. pulcherrima* crude extract against pathogenic bacteria, especially *A. baumannii* (8). Further purification of the partially purified fraction isolated from the crude extract is essential to obtain a more potent bioactive fraction. This should be accompanied by elucidation of the molecular mechanisms underlying its antimicrobial activity and the adaptive proteomic response to support the safe and effective development of natural compounds as promising antimicrobial agents.

Label-free quantitative (LFQ) LC-MS/MS proteomics provides a powerful and unbiased approach for characterizing global bacterial responses to antimicrobial stressors at the protein level (9). Conventional statistical approaches for proteomics, such as the standard Welch’s *t*-test, are susceptible to inflated false-positive rates when applied to data sets with few replicates (*n* = 3) and missing values, which are inherent features of LFQ data. Differential expression of quantified mass spectrometry data (DEqMS), developed by Zhu et al. (10), extends the limma empirical Bayes framework by incorporating peptide spectral count as a protein-specific weight for variance estimation. This approach assigns tighter variance estimates to proteins quantified by multiple peptides, providing improved statistical power and false discovery rate (FDR) control compared to limma alone or standard *t*-tests, particularly in small-n proteomics studies.

In our study, we partially purified a potential fraction from *C. pulcherrima* crude extract and evaluated the antimicrobial properties of the *C. pulcherrima* fraction against *A. baumannii* in both sensitive and multidrug-resistant strains. We further elucidated the molecular mechanisms responsible for the anti-MDR *A. baumannii* activity of CPF4 using LFQ LC-MS/MS proteomics and employed DEqMS-based LFQ LC-MS/MS proteomics to characterize the time-resolved proteomic response of *A. baumannii* (24, 48, and 72 h vs untreated control). We identified significantly differentially expressed proteins (DEPs) and performed integrative functional analyses encompassing gene ontology (GO) enrichment, time-series expression clustering, co-expression network analysis, and Virulence Factor Database (VFDB)/Comprehensive Antibiotic Resistance Database (CARD) virulence and resistance cross-referencing. Our findings reveal a temporally structured proteomic adaptation caused by a partially purified fraction from the *C. pulcherrima* yellow flower.

## MATERIALS AND METHODS

### Plant materials

Fresh yellow peacock flowers (*Caesalpinia pulcherrima*) were collected from Hatyai, Songkhla, during May to August 2024. Plant material was authenticated by a botanist at the Department of Biology, Faculty of Science, Prince of Songkla University, and a voucher specimen (WU-CP-2023-001) was deposited in the university herbarium. Fresh plant material (500 g) was washed with distilled water; the samples were dried in an oven at 60°C for a day and ground to a fine powder using a mechanical grinder. The powder was stored at −20°C until use.

### Sequential crude extraction and hexane, ethyl acetate, and ethanol-based isolation

The ground powder of *C. pulcherrima* flowers was sequentially extracted using *n*-hexane, ethyl acetate, and ethanol. Only the ethyl acetate fraction, which exhibited the strongest antibacterial activity, was selected for further purification. The extract was filtered, and the solvent was evaporated under reduced pressure using a rotary evaporator (Heidolph Laborota 4000, Germany); the resulting residue was stored at −20°C until further use.

The concentrated residue of the ethyl acetate extract was subjected to silica gel column chromatography using ethyl acetate/hexane (1:5 [vol/vol]) as the mobile phase. Thin layer chromatography analysis identified five major bands, which were individually collected based on their retention factor (*R_f_*) values (0.86, 0.71, 0.52, 0.24, and 0.13, respectively; developing solvent: ethyl acetate/hexane, 1:10 [vol/vol]). Each fraction, however, comprised a mixture of compounds with similar chromatographic behavior rather than a single pure compound. Solvents were removed under reduced pressure to afford the corresponding fractions. Fraction 4, which yielded the greatest quantity of precipitate, was selected for subsequent experiments examining the time-resolved proteomic response of *A. baumannii*. Fraction 4 of the *C. pulcherrima* ethyl acetate extract was hereafter referred to as CPF4.

### UPLC-MS chemical characterization

Untargeted metabolomic profiling of CPF4 was carried out using a Vanquish Flex UHPLC system (Thermo Scientific, Germering, Germany) coupled to an Orbitrap Exploris 120 mass spectrometer equipped with a heated electrospray ionization source and hybrid quadrupole-Orbitrap analyzer (Thermo Scientific, Bremen, Germany). Chromatographic separation was performed on a Hypersil Gold C18 column (2.1 × 150 mm, 1.9 µm; Thermo Fisher Scientific Inc., Waltham, MA, USA). For sample preparation, 10 mL of CPF4 solution (10 mg/mL in 60% methanol) was transferred into a 15 mL centrifuge tube, ultrasonicated at 25°C for 30 min, and centrifuged at 4,000 rpm for 10 min. The supernatant was then filtered through a 0.22 µm membrane filter prior to ultra-performance liquid chromatography coupled with mass spectrometry (UPLC-MS) analysis. The column temperature was maintained at 30°C, and the flow rate was set at 0.3 mL/min. Mobile phase A consisted of 0.1% formic acid in deionized water, while mobile phase B consisted of 0.1% formic acid in methanol. The gradient elution program was as follows: 10% B (0–5 min), 40% B (5–10 min), 50% B (10–20 min), and 100% B (20–25 min), followed by re-equilibration to 10% B (26–30 min). The total run time was 30 min with an injection volume of 5 µL. Mass spectrometric analysis was performed in full-scan mode using the following conditions: positive ion voltage 3,500 V, negative ion voltage 1,450 V, ion transfer tube temperature 325°C, vaporizer temperature 350°C, scan range m/z 100–1,000, and resolution 60,000. Raw UHPLC-MS data were processed using Compound Discoverer software version 3.3 (Thermo Fisher Scientific, USA). Putative metabolite annotation was performed based on accurate mass matching and database comparison against mzCloud (https://www.mzcloud.org) and ChemSpider (http://www.chemspider.com) databases. As authentic standards and MS/MS confirmation were not employed in the present study, the reported metabolite identities should be regarded as tentative annotations rather than definitive compound identifications.

### Bacterial strains and culture conditions

Two bacterial strains, *Acinetobacter baumannii* ATCC 19606 and an MDR *A. baumannii* clinical isolate, were obtained from the Department of Microbiology, School of Medicine, Walailak University, Thailand.

Overnight cultures were prepared in Mueller–Hinton (MH) broth (Gibco; Thermo Fisher Scientific, Inc.) at 37°C and subsequently adjusted to approximately 8 × 10⁸ CFU/mL before use in antimicrobial susceptibility testing. Gentamicin (Panreac AppliChem; ITW reagents) served as the positive control for both strains.

### Overlay spot assay for antimicrobial screening and microdilution method for minimum inhibitory concentration and minimum bactericidal concentration determination

Antimicrobial screening was performed following a modified protocol from Sirisarn et al. (11). Briefly, bacterial cultures grown overnight in MH broth at 37°C were diluted to 10^7^ CFU/mL and spread onto MH agar plates in three directions to achieve uniform lawns. The CPF4 was prepared at 10 mg/mL in dimethyl sulfoxide (DMSO) and sterilized with a 0.22 μm polytetrafluoroethylene filter. Subsequently, 10 μL of extract was spotted onto the inoculated plates, which were incubated at 37°C overnight. The presence of clear inhibition zones on the bacterial lawn was recorded as an indication of antimicrobial activity.

The broth microdilution procedure for determining minimum inhibitory concentration (MIC) and minimum bactericidal concentration (MBC) was adapted from Sirisarn et al. (11) (For MIC determination, 100 μL of MH broth was dispensed into each well of a sterile 96-well microplate. The CPF4 was initially diluted to 128 mg/mL in MH broth and subjected to twofold serial dilutions across the wells, producing concentrations ranging from 64 mg/mL down to 31.25 μg/mL. Then, 100 μL of bacterial suspension (10^6^ CFU/mL) was added to each well containing diluted CPF4, resulting in a final bacterial concentration of 5 × 10^5^ CFU/mL. As a growth control, 1% DMSO in MH broth was included in parallel. Plates were incubated at 37°C, and the MIC was defined as the lowest compound concentration that produced complete inhibition of visible bacterial growth.

To establish the MBCs, 10 μL aliquots from wells at and above the MIC were inoculated onto MH agar plates and incubated at 37°C. The MBC was defined as the lowest concentration of compound that resulted in the absence of visible bacterial colonies.

### Proteomic sample preparation and LC-MS/MS analysis

MDR *A. baumannii* (clinical isolates) was cultured overnight in MH broth at 37°C with shaking at 200 rpm. Cultures were diluted to OD_600_ = 0.1 in fresh MH broth and treated with a CPF4 fraction at a sub-lethal concentration (6,400 μg/mL; 0.25 × MIC). Cultures were then continuously incubated at 37°C with shaking, and samples were collected at 24 h, 48 h, and 72 h post-treatment, with three biological replicates per timepoint (*n* = 3; total 12 samples). An equivalent volume of DMSO, used to dissolve the CPF4 fraction, was included as a solvent control.

Bacterial cells equivalent to 1 OD_600_ unit were harvested at each time point by centrifugation at 10,000 × *g* for 5 min and washed twice with 1 mL of ddH_2_O. The cell pellet was resuspended in 100 μL of 0.5% (wt/vol) sodium dodecyl sulfate (SDS), and proteins were precipitated by the addition of ice-cold acetone at a 1:2 (vol/vol) ratio, followed by incubation at −80°C overnight. Precipitated proteins were recovered by centrifugation at 10,000 × *g* for 15 min, and the resulting pellet was resuspended in 0.5% (wt/vol) SDS. Protein concentration was determined using the Lowry assay (12), with bovine serum albumin as the standard.

Five micrograms of total protein from each sample were subjected to in-solution digestion. Samples were dissolved in 10 mM ammonium bicarbonate, reduced with 5 mM dithiothreitol at 60°C for 1 h, and alkylated with 15 mM iodoacetamide at room temperature for 45 min in the dark. Sequencing-grade trypsin (Promega, Madison, WI, USA) was added at an enzyme-to-substrate ratio of 1:20 (wt/wt), and digestion was carried out at 37°C for 16 h. The resulting peptide mixtures were dried under vacuum and reconstituted in 0.1% (vol/vol) formic acid prior to LC-MS/MS analysis.

Tryptic peptides were separated and analyzed using an Ultimate 3000 Nano/Capillary LC System (Thermo Scientific, Waltham, MA, USA) coupled to a ZenoTOF 7600 mass spectrometer (SCIEX, Framingham, MA, USA). Data were acquired in data-dependent acquisition mode, in which the top 50 most abundant precursor ions identified per MS1 survey scan were selected for fragmentation at an intensity threshold exceeding 150 counts per second. Selected precursor ions were dynamically excluded for 12 s following two successive MS/MS sampling events, with dynamic collision energy enabled for MS/MS. MS2 spectra were collected over a mass range of 100–1,800 *m/z* with an accumulation time of 50 ms, with the Zeno trap enabled to enhance sensitivity. To minimize experimental variation, three independent LC-MS/MS runs were performed for each sample.

MaxQuant version 2.5.0.0 (13) was used to quantify and identify the proteins in individual samples using the Andromeda search engine to correlate raw MS/MS spectra to the known proteins related to *A. baumannii* downloaded from the Uniprot *Acinetobacter baumannii* database (17 March 2025). The MaxQuant’s standard setting parameters used were enzyme (trypsin), fixed modification (carbamidomethylation of cysteine residues), variable modifications (oxidation of methionine residues and acetylation of the protein N-terminus), and two missed cleavages. Protein FDR was set at 1% and estimated by the reverse search sequences.

### Data pre-processing and quality control

**Fig 1:**
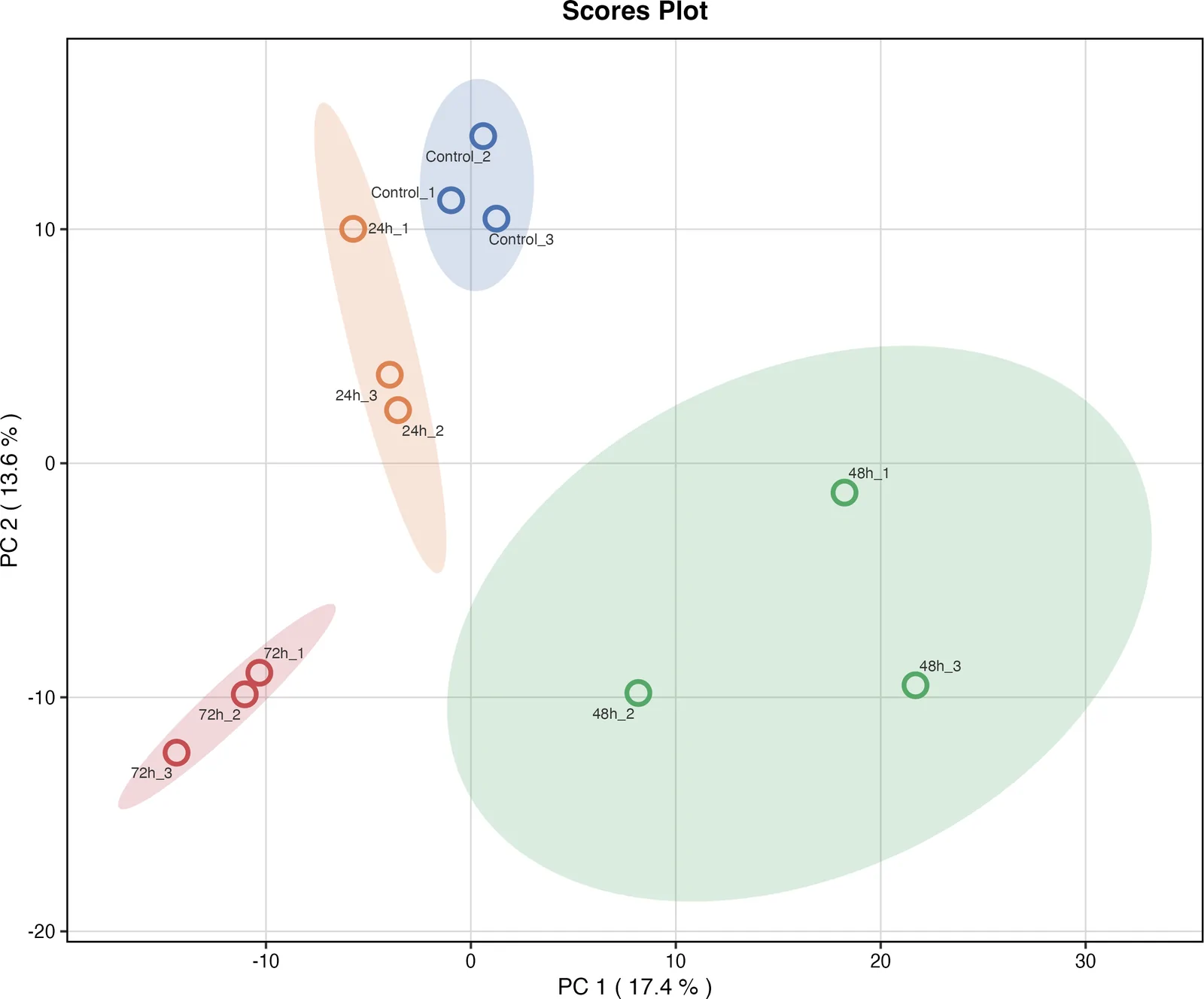
Principal component analysis (PCA) scores plot of all 12 samples based on the full imputed log₂ intensity matrix. Each point represents one biological replicate. Shaded ellipses indicate 95% confidence regions per group (computed by eigendecomposition of group covariance matrices). Hollow circles with colored borders denote individual samples. PC1 = 17.4% variance; PC2 = 13.6% variance.

**Fig 2:**
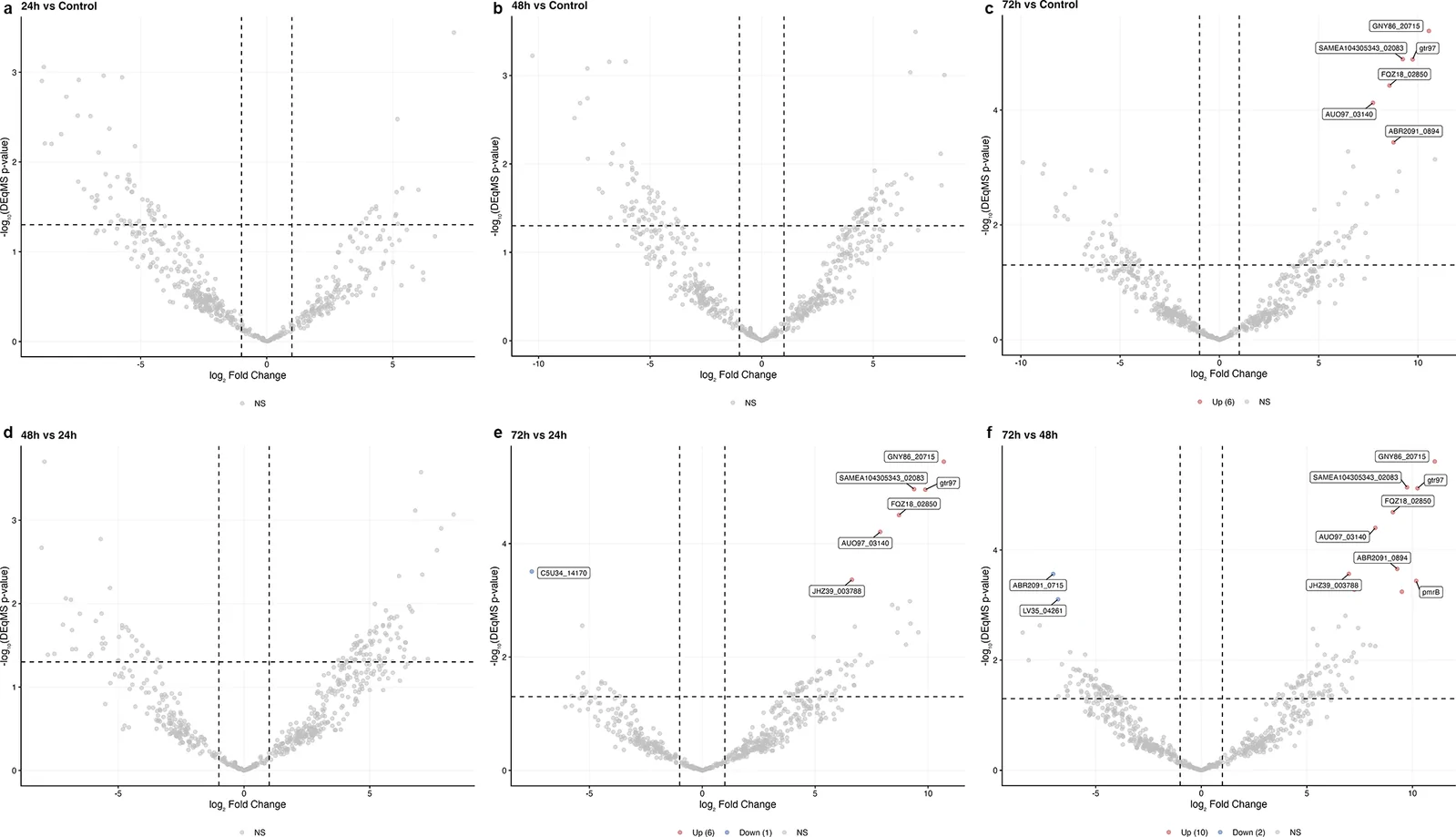
**(a–f)** DEqMS volcano plots for six pairwise comparisons: (**a**) 24 h vs Control, (**b**) 48 h vs Control, (**c**) 72 h vs Control, (**d**) 48 h vs 24 h, (**e**) 72 h vs 24 h, and (**f**) 72 h vs 48 h. X-axis: log₂ fold change; y-axis: −log_10_(DEqMS *P*-value). Dashed horizontal line: nominal *P* = 0.05 threshold. Dashed vertical lines: |log_2_FC| = 1. Red: upregulated DEPs; blue: downregulated DEPs; gray: non-significant (NS). Top-ranked proteins labeled by gene name or UniProt locus tag.

Proteins were retained if detected in ≥2 of three biological replicates in at least one experimental group, yielding 715 high-confidence proteins for quantitative analysis (Fig. 1). LFQ intensities were on a log₂ scale. Missing values were imputed using a half-minimum per-column approach, appropriate for values missing-not-at-random in LFQ data. Data quality and inter-sample reproducibility were assessed by principal component analysis (PCA) of the full imputed intensity matrix (Fig. 2).

### DEqMS differential expression analysis

Differential protein expression was assessed using the DEqMS R package (v1.12) [10], which extends the limma empirical Bayes framework by weighting variance estimation according to the number of peptides quantified per protein. Briefly, a linear model was fitted per protein using the limma *lmFit*() function with a no-intercept design matrix across the four groups. Pairwise contrasts for all six comparisons were computed using *makeContrasts*(), followed by *eBayes*() for initial moderated t-statistics. Peptide spectral counts were subsequently incorporated, and *spectraCounteBayes*() was applied to compute DEqMS posterior variances and moderated t-statistics (sca.t). Results for each contrast were extracted using *outputResult*(). Multiple testing correction was performed using the Benjamini–Hochberg FDR (BH-FDR) method. Proteins were considered differentially expressed at |log₂FC| ≥ 1 and a DEqMS-adjusted *P*-value < 0.05. Additionally, an *F*-test applied simultaneously across all four groups (ANOVA-equivalent) was used to identify proteins with significant temporal variation.

### GO over-representation analysis

GO over-representation analysis was performed using Fisher’s exact test (one-sided, alternative = “greater”) for the biological process (BP), cellular component, and molecular function (MF) ontologies, with GO annotations sourced from UniProtKB. The background gene set comprised all 715 detected proteins. BH-FDR correction was applied to all resulting *P*-values. Results were visualized as bubble dot plots, in which bubble size is proportional to the number of significantly DEPs, and color intensity represents −log_10_(adjusted *P*-value). Terms passing FDR correction (adjusted *P* < 0.05) are indicated by a star symbol (★).

### KEGG pathway enrichment

KEGG pathway over-representation analysis was performed using a curated gene set encompassing 21 pathways relevant to *A. baumannii* biology, including central metabolism, biosynthesis, genetic information processing, cell envelope biogenesis, antimicrobial resistance, and virulence. Gene-to-pathway membership was assigned based on gene name matching against the KEGG pathway database. Fisher’s exact test (one-sided, alternative = “greater”) with BH-FDR correction was applied for each pairwise comparison.

### Time-series cluster analysis

All 715 detected proteins were subjected to *k*-means clustering (*k* = 6, determined by the elbow criterion) based on their *Z*-score-normalized mean expression profiles across the four experimental groups (Control, 24 h, 48 h, and 72 h). Cluster expression trajectories were characterized and labeled according to their dominant temporal expression pattern. To visualize global expression trends, the 150 highest-variance proteins were displayed as a cluster-sorted heatmap.

### Co-expression network analysis

A co-expression network was constructed for all significantly DEPs (derived from the union of all pairwise comparisons) using Pearson correlation coefficients (*r* ≥ 0.85) calculated across group mean expression values. Network visualization was performed using the Fruchterman–Reingold force-directed layout algorithm as implemented in the igraph R package. Node size was scaled according to degree centrality, and node color indicates the direction of regulation at the most extreme pairwise comparison. Additionally, STRING-compatible protein lists were exported for subsequent protein–protein interaction network analysis.

### Virulence and resistance cross-referencing

All 715 detected proteins were cross-referenced against curated gene lists derived from the VFDB (10 virulence factor categories) and the CARD (seven resistance mechanism categories) using gene name matching. Proteins were subsequently annotated as either detected or statistically significant (DEqMS-adjusted *P* < 0.05). Proteins meeting the nominal significance threshold (*P* < 0.05) but failing to survive BH-FDR correction were additionally flagged to distinguish them from those meeting the stringent significance criterion.

### Software

All statistical analyses and visualizations were performed in R (v4.3.x) using the following packages: DEqMS (v1.12), limma (v3.56), ggplot2 (v3.4), ggrepel, pheatmap, igraph, dplyr, and openxlsx. PCA was computed using the *prcomp*() function with mean centering and unit variance scaling applied prior to decomposition. Group-level 95% confidence ellipses were derived by eigendecomposition of the group covariance matrices, assuming a chi-squared distribution with two degrees of freedom (*χ*² df = 2).

## RESULTS

### Chemical characterization of *C. pulcherrima* fraction

The dried powder of CPF4, which appeared dark green in color (Fig. S1 available at https://doi.org/10.5281/zenodo.21543825) was dissolved in 60% (vol/vol) methanol and subjected to untargeted metabolomic profiling by UPLC-MS. UPLC-MS profiling revealed that CPF4 contained a total of 71 compound entries, a chemically diverse mixture of lipid-associated, phenolic, alkaloid-like, and amphiphilic compounds (Table S1 available at https://doi.org/10.5281/zenodo.21543825). Among the most abundant tentatively annotated metabolites were summarized in Table 1, ranked by maximum peak area. Of these, six compounds have been previously reported to possess antimicrobial properties. Oleamide (C_18_H_35_NO; peak area: 2.52 × 10^9^) was the most abundant compound detected. Kahweol (C_20_H_26_O_3_; peak area: 8.76 × 10^8^), a diterpene characteristic of coffee, ranked seventh in abundance. Docosahexaenoic acid (DHA; C_22_H_32_O_2_; peak area: 2.78 × 10^8^) is a polyunsaturated omega-3 fatty acid, while myricitrin (C_21_H_20_O_12_; peak area: 4.78 × 10^7^) is a flavonoid glycoside present at comparatively lower abundance. Trigonelline (C_7_H_7_NO_2_; peak area: 3.79 × 10^7^), a pyridinium alkaloid, and azelaic acid (C_9_H_16_O_4_; peak area: 1.92 × 10^8^), a dicarboxylic acid, were also identified. However, as these annotations were assigned mainly based on accurate mass and retention behavior, they should be considered provisional pending further structural confirmation. In addition to plant-associated metabolites, several detected signals corresponded to compounds frequently encountered as environmental or analytical contaminants, including phthalate derivatives and surfactant-related compounds. For this reason, interpretation focused more broadly on metabolite classes that have previously been linked to antimicrobial activity in plant extracts, rather than attributing the observed bioactivity to any single detected compound. Overall, the metabolite profile suggests that CPF4 is enriched in amphiphilic and phenolic-like constituents that may collectively contribute to membrane perturbation and stress induction in gram-negative bacteria. Nevertheless, the specific compounds responsible for the antibacterial activity observed in this study remain unclear and will require further purification and targeted bioactivity-guided investigation.

**TABLE 1:** Top 30 compounds identified in the CPF4 by UPLC-MS (ranked by maximum peak area; semi-quantified relative to vitexin internal standard)

Compound name	Formula	Calc. MW	m/z	RT (min)	Area (Max.)
Oleamide	C_18_H_35_NO	281.27	282.28	26.84	2,518,586,362
Citroflex A-4	C_20_H_34_O_8_	402.23	425.21	25.78	1,436,813,830
(4S,5S,8S,10R)−4,5,8-trihydroxy-10-methyl hexahydrooxecin-2-one	C_10_H_16_O_5_	216.10	217.10	2.45	1,311,168,691
Diisobutylphthalate	C_16_H_22_O_4_	278.15	301.14	25.36	1,176,186,980
4-Dodecylbenzenesulfonic acid	C_18_H_30_O_3_S	326.19	325.19	26.99	889,678,019
Kahweol	C_20_H_26_O_3_	314.19	315.19	25.64	876,240,915
Veratrole	C_8_H_10_O_2_	138.07	139.08	12.57	782,977,524
Decanamide	C_10_H_21_NO	171.16	172.17	24.52	770,155,398
Bis(2-ethylhexyl) terephthalate	C_24_H_38_O_4_	390.28	391.28	25.75	582,590,032
Hexadecanamide	C_16_H_33_NO	255.26	256.26	26.74	445,130,885
Bis(2-ethylhexyl) phthalate	C_24_H_38_O_4_	390.28	391.28	25.98	419,268,278
Ostruthin	C_19_H_22_O_3_	298.16	297.15	24.63	342,143,812
Dodecyl sulfate	C_12_H_26_O_4_S	266.16	265.15	24.38	334,457,639
Docosahexaenoic acid (DHA)	C_22_H_32_O_2_	308.23	341.27	26.44	277,697,103
1,3,7-Trimethylxanthine derivative	C_8_H_10_N_4_O_2_	194.08	195.09	3.32	257,110,639
Palmitelaidic acid methyl ester	C_17_H_32_O_2_	268.24	269.25	25.37	239,828,341
Ethyl oleate	C_20_H_38_O_2_	310.29	311.29	26.10	223,693,707
19-Norandrostenedione	C_18_H_24_O_2_	272.18	273.18	25.52	202,913,196
Methyl palmitate	C_17_H_34_O_2_	287.28	288.29	24.46	194,932,583
Azelaic acid	C_9_H_16_O_4_	188.11	187.10	12.71	192,122,078
T-2 Triol	C_20_H_30_O_7_	360.21	383.20	25.44	167,842,872
1-Linoleoyl glycerol	C_21_H_38_O_4_	336.27	337.27	25.65	127,742,202
Olomoucine	C_15_H_18_N_6_O	276.17	299.16	24.67	114,173,502
Choline	C_5_H_13_NO	103.10	104.11	0.76	111,716,305
Myristyl sulfate	C_14_H_30_O_4_S	294.19	293.18	26.30	121,844,883
Myricitrin	C_21_H_20_O_12_	464.10	463.09	10.68	47,839,603
Trigonelline	C_7_H_7_NO_2_	137.05	138.05	0.84	37,853,775
α,α-Trehalose	C_12_H_22_O_11_	342.12	341.11	0.83	17,068,209
Heptadecanoic acid	C_17_H_34_O_2_	292.24	293.25	26.19	8,460,198
2,6-Xylidine	C_8_H_11_N	121.09	122.10	0.90	14,429,380

Analysis of CPF4 yielded a total of 71 compound entries, corresponding to approximately 45 unique chemical entities after accounting for multiple chromatographic peaks arising from the same compound (Table S1 available at https://doi.org/10.5281/zenodo.21543825). The identified compounds spanned diverse chemical classes, including fatty acid amides, diterpenes, polyunsaturated fatty acids, flavonoid glycosides, alkaloids, organic acids, phthalate esters, and sulfonic acids.

The 30 highest-abundance unique compounds are summarized in Table 1, ranked by maximum peak area. Of these, six compounds have been previously reported to possess antimicrobial properties. Notably, several high-abundance compounds—including phthalate esters (diisobutylphthalate, bis(2-ethylhexyl) phthalate, and bis(2-ethylhexyl) terephthalate), alkylbenzene sulfonic acids (4-dodecylbenzenesulfonic acid), and dodecyl sulfate—are likely process-related contaminants or instrument-derived plasticizers rather than genuine bioactive constituents of the extract and are therefore excluded from further discussion.

### Antimicrobial activity against *A. baumannii*

The fourth fraction of the *C. pulcherrima* ethyl acetate extract (CPF4) was screened for antimicrobial activity against *A. baumannii* ATCC19606 (a susceptible reference strain) and an MDR clinical isolate using overlay spot assay and broth microdilution methods. CPF4 (1.28 mg/spot) produced clear inhibition zones on bacterial lawns of both strains: *A. baumannii* ATCC19606 and the MDR strain, confirming antimicrobial activity (Table 2). Gentamicin (10 µg/mL) served as a positive control and produced inhibition zones against *A. baumannii* ATCC19606 but not against the MDR clinical isolate, confirming the multidrug-resistant phenotype of the latter. An equivalent volume of DMSO, included as a solvent control, produced no inhibition.

**TABLE 2:** Antimicrobial activity using an overlay spotted assay of yellow peacock flower extract screened against *A. baumannii* species^*a*^

Pathogenic isolates	Antimicrobial activity of yellow peacock flower extract (1.28 mg/spot)	Positive control (gen)	Negative control (1% DMSO)
*Acinetobacter baumannii* ATCC19606	+	+	−
Multidrug-resistant *Acinetobacter baumannii* (MDR)	+	−	−

^*a*^ A total of 10 µg/mL of gentamicin (gen) was used as a positive control for gram-negative bacteria. DMSO was used as a negative control. Symbols + and – indicate the presence and absence of a clear zone on the bacterial lawn, respectively. All tests were conducted in triplicate.

The MIC and MBC of CPF4 were subsequently determined by broth microdilution. CPF4 exhibited potent activity against *A. baumannii* ATCC19606, with both MIC and MBC values of 31.25 µg/mL, indicating that the bactericidal concentration coincided with the MIC. In contrast, CPF4 showed no appreciable activity against the MDR clinical isolate, with MIC and MBC values both exceeding 64,000 µg/mL, representing a greater than 2,000-fold difference in susceptibility between the two strains (Table 3). Collectively, these results demonstrate that the CPF4 possesses potent antimicrobial properties against susceptible *A. baumannii* but is ineffective against the MDR clinical isolate. To elucidate the molecular response of the MDR strain to CPF4 exposure, a time-resolved proteomic analysis was performed at 24 h, 48 h, and 72 h post-treatment.

**TABLE 3:** Minimum inhibitory concentrations and minimum bactericidal concentrations of yellow peacock flower extract against *Acinetobacter baumannii* species^*a*^

Pathogenic isolates	MIC/MBC (mg/mL) yellow peacock flower extract	MIC (µg/mL) gentamicin	Negative control (1% DMSO)
*Acinetobacter baumannii* ATCC19606	0.03125/0.03125	<0.03125	−
Multidrug-resistant *Acinetobacter baumannii* (MDR)	>64/>64	>64	−

^*a*^ Gentamicin (gen) was used as a positive control for the *Acinetobacter baumannii* species. DMSO (1%) was used as a negative control diluted in MHB broth. All tests were conducted in triplicate.

### Data quality and sample overview

The MDR *A. baumannii* clinical isolate was cultured in MH broth and adjusted to an optical density of OD_600_ = 0.1. CPF4 was then added to the culture at a sub-lethal concentration of 6,400 µg/mL. Cultures were harvested at 24 h, 48 h, and 72 h post-treatment and subjected to LC-MS/MS-based proteomics analysis. Proteomic profiling across 12 samples yielded 3,414 protein group identifications. Following quality filtering, 715 proteins (20.9%) were retained for downstream analysis, constituting the high-confidence quantitative data set. Per-sample detection rates ranged from approximately 477–621 proteins per replicate across experimental groups (Fig. S2a available at https://doi.org/10.5281/zenodo.21543825), with consistent log_2_ intensity distributions observed across all samples (Fig. S2b available at https://doi.org/10.5281/zenodo.21543825), collectively confirming adequate analytical performance. The transient reduction in the number of detected proteins at the 24 h time point relative to the control is consistent with an initial suppression of bacterial protein synthesis or reduced metabolic activity in response to acute CPF4 exposure.

PCA of the full imputed intensity matrix revealed tight clustering of biological replicates within each experimental group (Fig. 1). The four treatment conditions separated clearly along the principal axes, with PC1 explaining 17.4% and PC2 explaining 13.6% of the total variance. Notably, replicates from the 48 h treatment group exhibited greater intra-group dispersion relative to the 24 h, 72 h, and control groups, suggesting increased inter-replicate variability in the proteomic response at this intermediate time point. Overall, the clear separation between treated and control conditions in PCA space supports the validity of the experimental design and is consistent with a CPF4-driven proteomic reprogramming of the MDR *A. baumannii* proteome.

### Differential expression analysis

To identify DEPs, DEqMS analysis was performed, revealing a total of 25 unique significant DEPs across all six pairwise comparisons (|log_2_FC| ≥ 1, BH-adjusted DEqMS *P*-value < 0.05; Fig. 2a through f; Table S1 available at https://doi.org/10.5281/zenodo.21543825). The distribution of DEPs across comparisons is summarized in Table 4. No significant DEPs were detected at either 24 h vs Control (0 DEPs; Fig. 2a) or 48 h vs Control (0 DEPs; Fig. 2b) comparisons, indicating that CPF4 treatment did not elicit detectable proteome-level changes during the first 48 h at the applied significance threshold. In contrast, the 72 h vs Control comparison yielded six upregulated DEPs with no downregulated DEPs (Fig. 2c), suggesting that significant proteomic remodeling in the MDR *A. baumannii* occurs primarily at the 72 h time point.

**TABLE 4:** DEqMS significantly upregulated proteins at 72 h vs control

Entry	Gene/ID	Protein name	log₂FC	Adjusted *P* (DEqMS)	*n* pep
A0A6G0V7P4	GNY86_20715	Helix-turn-helix domain-containing protein	+10.55	0.003	1
A0A6B9KUR5	gtr97	Glycosyltransferase Gtr97	+9.73	0.003	1
A0AB38GLP2	SAMEA104305343_02083	Uncharacterized protein	+9.23	0.003	1
A0A7U7Q8J5	ABR2091_0894	DNA helicase	+8.77	0.043	1
A0AAN5W9Y9	FQZ18_02850	Uncharacterized protein	+8.57	0.007	1
A0A1E3MBP3	AUO97_03140	3-methyladenine DNA glycosylase	+7.73	0.011	1

The six proteins significantly upregulated at 72 h relative to the control are detailed in Table 4. The most significantly upregulated protein was GNY86_20715 (log_2_FC = +10.55, adjusted *P* = 0.003), annotated as a helix-turn-helix (HTH) domain-containing protein, a structural motif characteristic of DNA-binding transcriptional regulators. The glycosyltransferase Gtr97 (log_2_FC = +9.73, adjusted *P* = 0.003) and an uncharacterized protein, SAMEA104305343_02083 (log_2_FC = +9.23, adjusted *P* = 0.003), also exhibited among the highest-magnitude inductions. Additionally, a putative 3-methyladenine DNA glycosylase (AUO97_03140; log_2_FC = +7.73, adjusted *P* = 0.011) and a DNA helicase (ABR2091_0894; log_2_FC = +8.77, adjusted *P* = 0.043) were upregulated, collectively implicating the activation of DNA damage responses.

Between-timepoint comparisons further revealed the temporal dynamics of proteomic changes. The 72 h vs 48 h comparison yielded the largest DEP set (12 DEPs total: 10 upregulated and 2 downregulated; Fig. 2f), indicating an escalating adaptive response between the mid- and late-treatment phases. Additional proteins upregulated in this comparison included Sel1 repeat family protein JHZ39_003788 (log_2_FC = +6.99, adjusted *P* = 0.024), the histidine kinase PmrB (log_2_FC = +10.18, adjusted *P* = 0.029), the glycosyltransferase Gtr162 (log_2_FC = +9.50, adjusted *P* = 0.037), and uncharacterized protein MKP18_001899 (log_2_FC = +7.25, adjusted *P* = 0.037). Two proteins were significantly downregulated at the 72 h vs 48 h comparison: putative membrane protein ABR2091_0715 (log_2_FC = −7.02, adjusted *P* = 0.024) and HNH endonuclease LV35_04261 (log_2_FC = −6.79, adjusted *P* = 0.047).

The 72 h vs 24 h comparison revealed seven DEPs (six upregulated and one downregulated). The sole downregulated protein, C5U34_14170 (log_2_FC = −7.55, adjusted *P* = 0.037), is consistent with its transient peak expression observed at 24 h (Fig. 4). Volcano plots for all six comparisons are presented in Fig. 2a through f.

**Fig 3:**
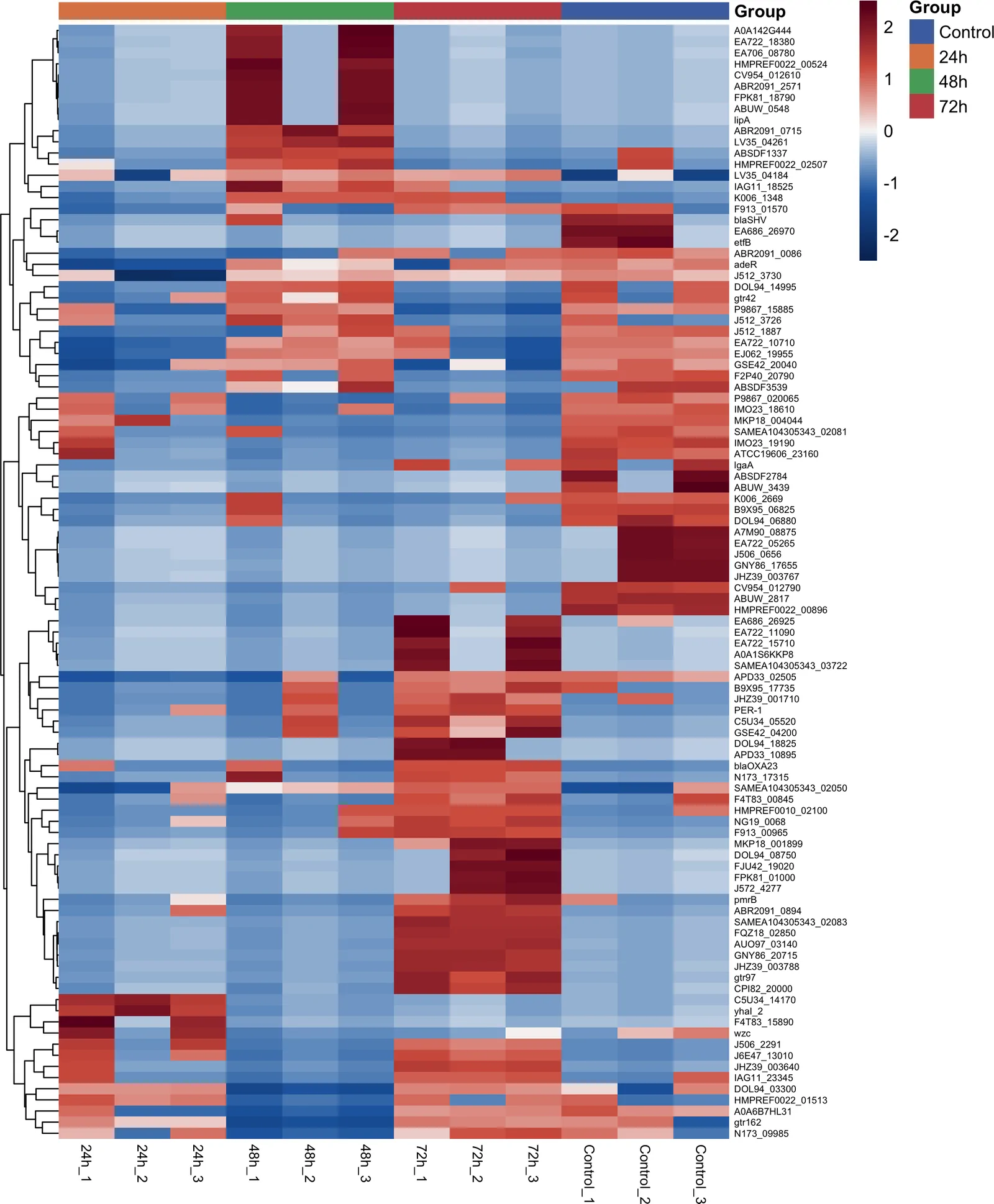
Heatmap of top 100 ANOVA-equivalent significant proteins (ranked by DEqMS *F*-test adjusted *P*-value). Rows = proteins with hierarchical clustering; columns = 12 samples grouped by treatment. Color scale = row-wise *Z*-scored log_2_ intensity (red = high and blue = low). Group color bars at the top indicate treatment condition.

**Fig 4:**
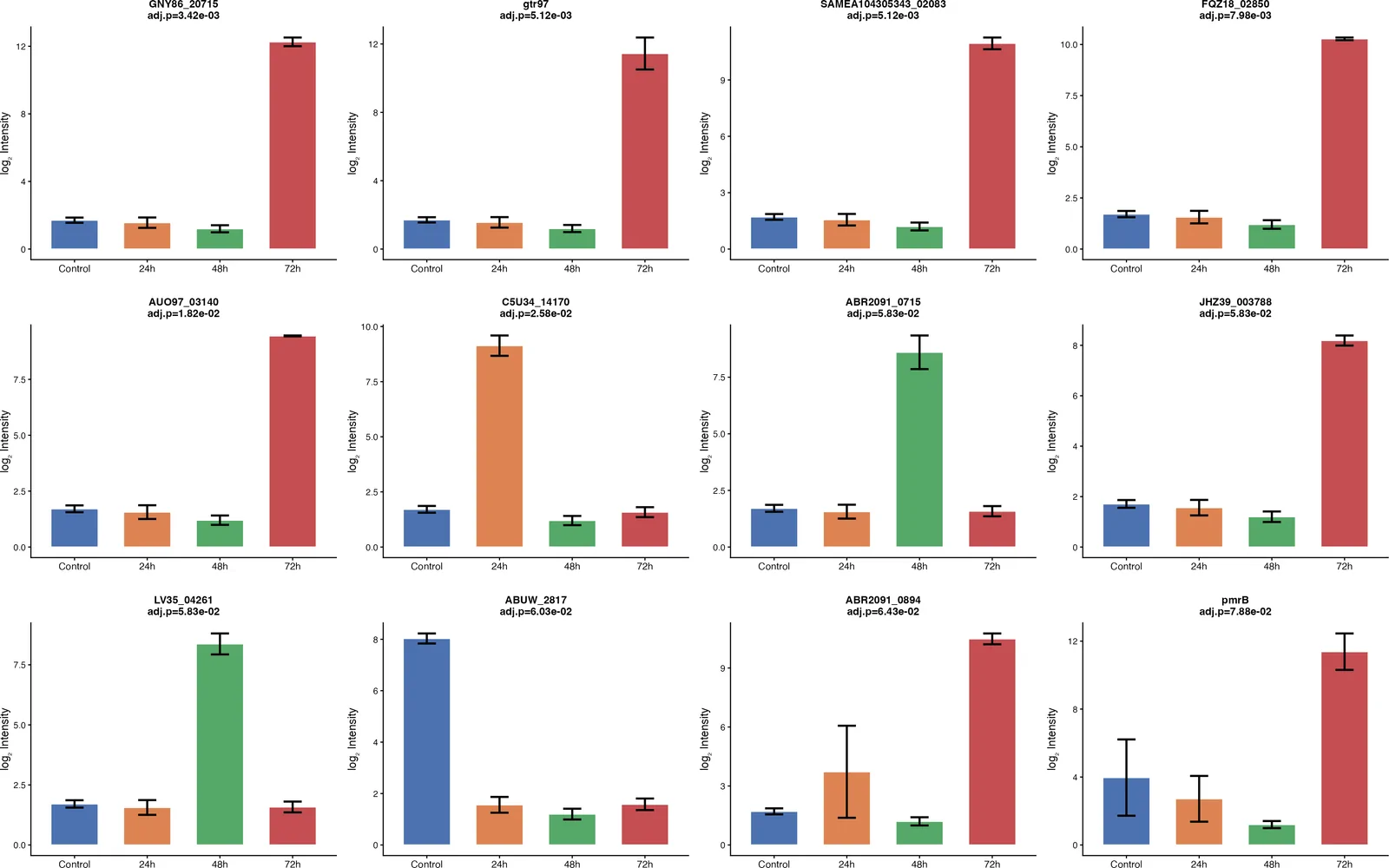
Expression profiles of the top 12 ANOVA-significant proteins. Each panel shows mean log_2_ LFQ intensity ± SEM across the four experimental groups. DEqMS *F*-test adjusted *P*-values are indicated in each panel title. Bar color corresponds to the treatment group.

ANOVA applied simultaneously across all four groups (Fig. 3) confirmed the temporal dynamics of protein expression, with the most significantly varying proteins largely consistent with the late-upregulated candidates identified in the pairwise comparisons. Figure 4 displays the expression trajectories of the 12 most ANOVA-significant proteins, illustrating a dominant pattern of near-baseline expression at 24 h and 48 h followed by marked induction at 72 h for the HTH domain-containing protein, glycosyltransferase Gtr97, 3-methyladenine DNA glycosylase, DNA helicase, and two uncharacterized proteins (SAMEA104305343_02083 and FQZ18_02850). In contrast, uncharacterized protein C5U34_14170 exhibited a distinct pattern of early induction at 24 h, followed by a return to near-baseline levels at subsequent time points. The uncharacterized protein ABUW_2817 displayed comparatively elevated expression in the control that declined progressively over the course of CPF4 treatment, suggesting suppression of this protein under the experimental conditions applied.

### GO over-representation analysis

**Fig 5:**
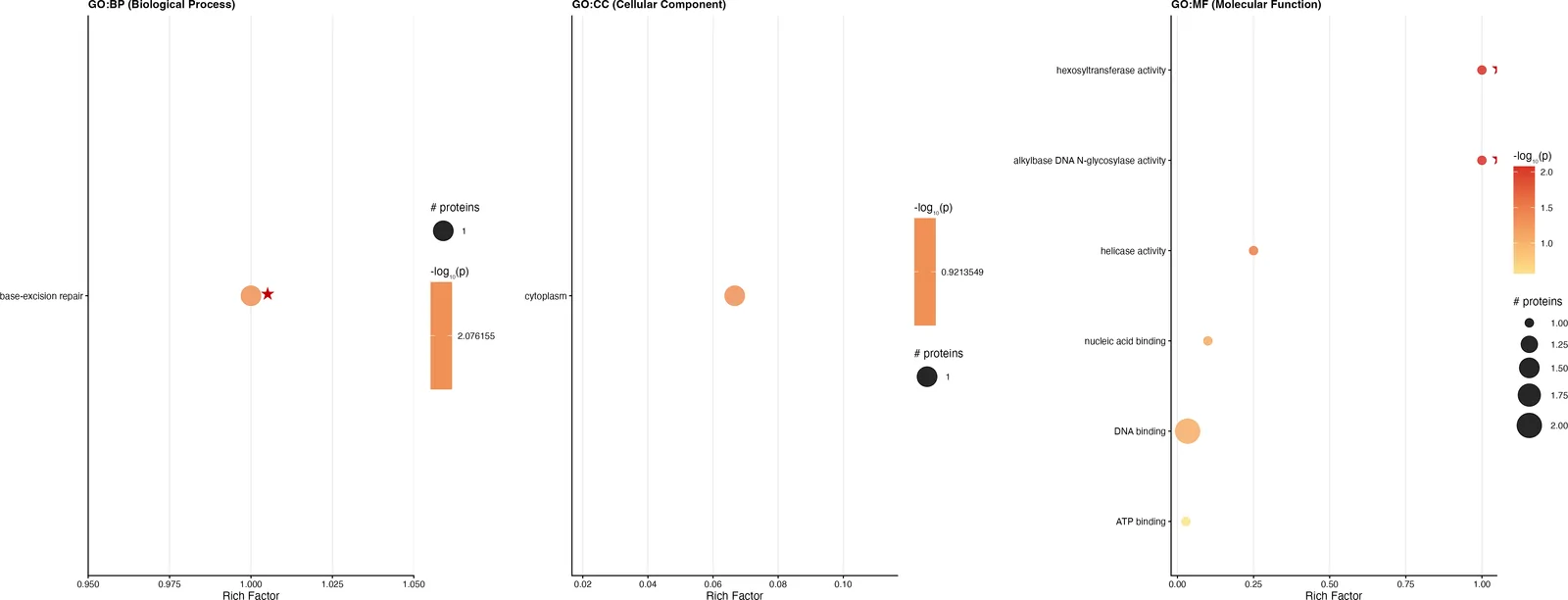
GO over-representation analysis for the 72 h vs Control comparison across three ontology domains: biological process (BP; left), cellular component (CC; center), and molecular function (MF; right). Foreground = 6 upregulated DEPs; background = 715 detected proteins. Bubble size = number of DEPs annotated to each term. Color intensity = −log_10_(*P*-value). ★ = FDR-corrected adjusted *P* < 0.05. Top 15 terms shown per ontology.

GO over-representation analysis, conducted using the six significantly upregulated DEPs at 72 h vs Control as the foreground and all 715 detected proteins as the background, identified three functionally significant enrichments (Fig. 5). In the biological process (GO:BP) ontology, base-excision repair (BER) was the sole significantly enriched term (rich factor = 1.00, FDR adjusted *P* < 0.05, ★; Fig. 5, left panel). This enrichment is supported by the marked induction of 3-methyladenine DNA glycosylase (AUO97_03140), a key enzyme in the BER pathway responsible for removing alkylated DNA bases generated by reactive electrophiles and oxidative stress. The detection of this enrichment at 72 h, and not at earlier time points, indicates that BER induction is a late-phase response to treatment, consistent with progressive accumulation of oxidative or alkylating DNA damage under sustained CPF4 exposure.

**Fig 6:**
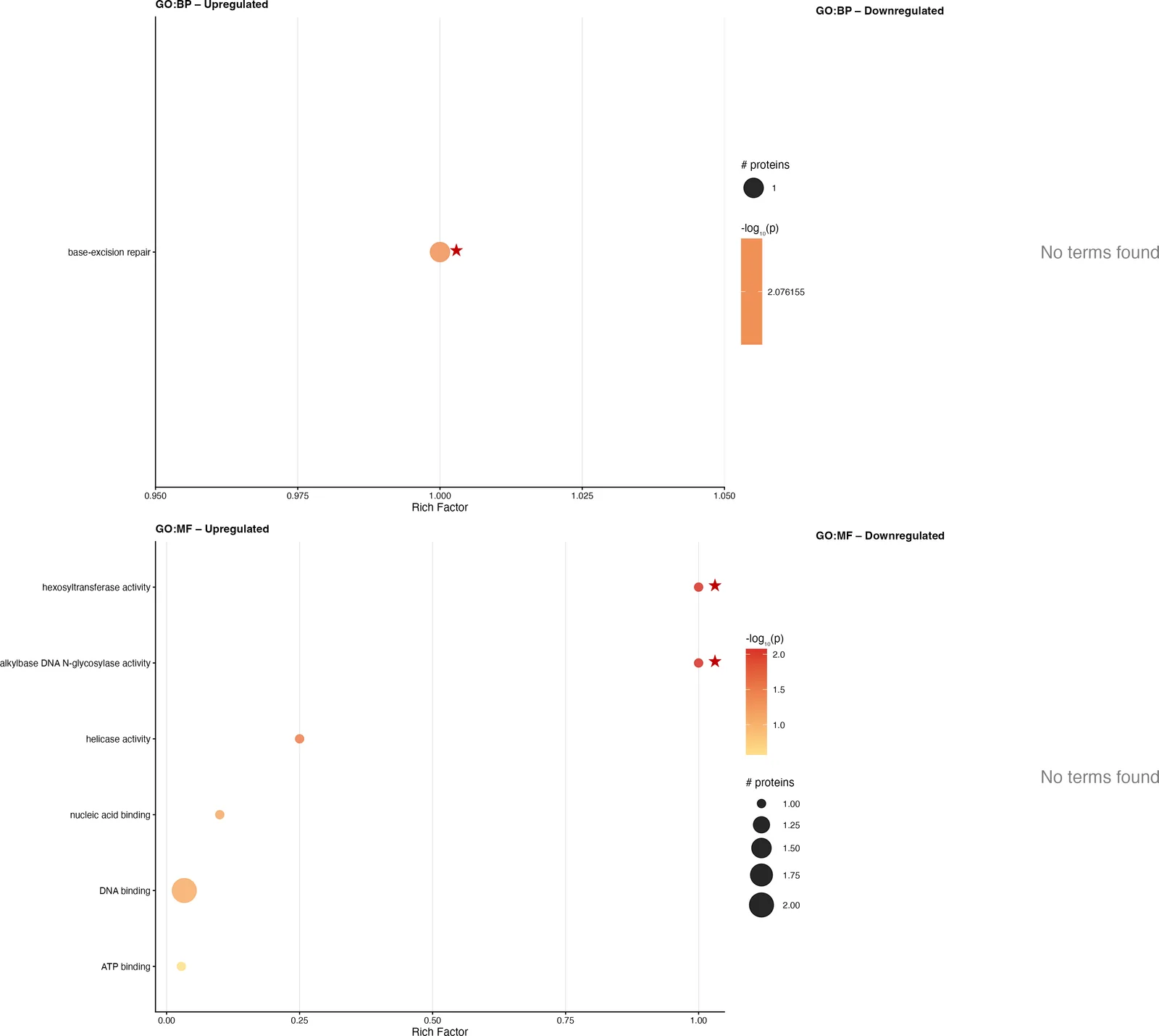
Directional GO enrichment at 72 h vs Control. Top row: GO:BP enrichment in upregulated (left) and downregulated (right) DEPs. Bottom row: GO:MF enrichment in upregulated (left) and downregulated (right) DEPs. ★= FDR adjusted *P* < 0.05. No significant terms were identified in the downregulated direction.

In the molecular function (GO:MF) ontology, two terms reached FDR significance (Fig. 5, right panel; Fig. S4 available at https://doi.org/10.5281/zenodo.21543825): hexosyltransferase activity (rich factor = 1.00, ★) and alkylbase DNA N-glycosylase activity (rich factor = 1.00, ★). Hexosyltransferase activity was attributable to the induction of glycosyltransferase Gtr97 and the GNY86_20715 locus, suggesting active remodeling of surface polysaccharides or lipopolysaccharide (LPS) structures. Alkylbase DNA N-glycosylase activity was driven by AUO97_03140, consistent with the BER enrichment identified in GO:BP. Additional nominally enriched GO:MF terms included helicase activity (attributable to the DNA helicase ABR2091_0894), nucleic acid binding, DNA binding, and ATP binding (Fig. 6). Directional analysis confirmed that all significant GO:MF enrichments were confined to the upregulated DEP set at 72 h, with no significant terms detected among the downregulated direction (Fig. S4 available at https://doi.org/10.5281/zenodo.21543825), consistent with the absence of downregulated DEPs in this comparison. No GO terms reached FDR significance at 24 h vs Control or 48 h vs Control comparisons. KEGG pathway enrichment analysis did not identify any significantly enriched pathways, likely due to the limited number of DEPs relative to the gene set sizes of typical KEGG pathways.

### Time-series expression cluster analysis

**Fig 7:**
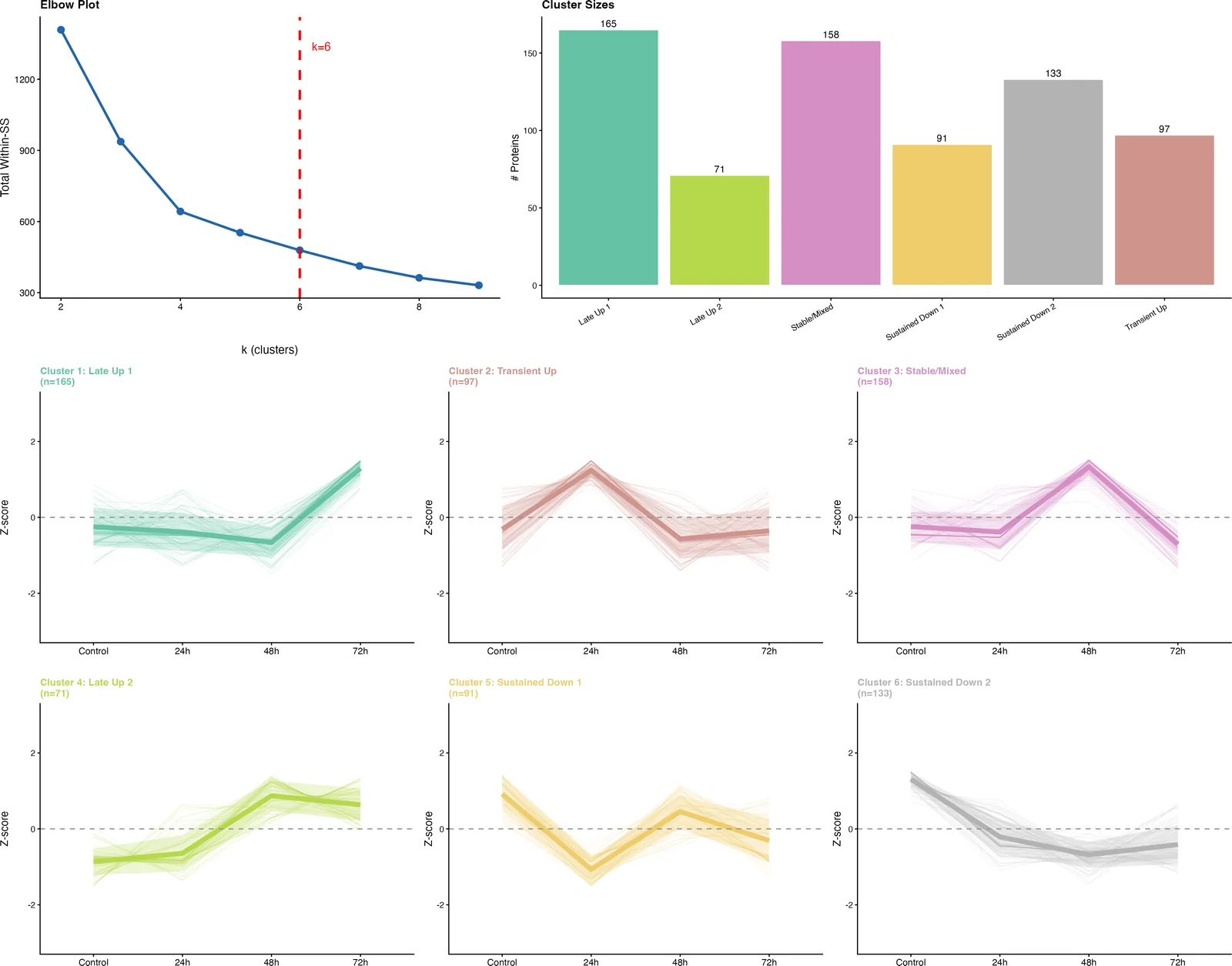
Time-series *k*-means cluster analysis (*k* = 6). Top left: Elbow plot of total within-cluster sum of squares vs *k*; red dashed line = selected *k* =6. Top right: Cluster size bar chart. Panels 1–6: Mean expression trajectories per cluster: bold line = cluster mean; faint lines = individual protein profiles (up to 80); shaded area = ±1 SD. All profiles are *Z*-score-normalized log_2_ intensities across Control, 24 h, 48 h, and 72 h.

*K*-means clustering (*k* = 6, determined by the elbow criterion) of all 715 proteins based on *Z*-score-normalized group mean expression profiles identified six biologically distinct trajectory classes (Fig. 7). Cluster sizes and trajectory descriptors are presented in Table 5. The two Late Up clusters (Cluster 1 and Cluster 4; combined *n* = 236 proteins) comprised the largest protein group, both exhibiting progressive induction peaking at 72 h, consistent with a sustained adaptive response to prolonged CPF4 exposure. All six significantly upregulated DEPs identified in the 72 h vs Control comparison were assigned to these clusters.

**TABLE 5:** Time-series *K*-means cluster summary (*k* = 6)

Cluster	Pattern	Control (*Z*)	24 h (*Z*)	48 h (*Z*)	72 h (*Z*)	*n* Proteins
1	Late up 1	~0.0	~−0.4	~0.0	+1.3	165
2	Transient up	~0.0	+1.2	~0.0	~−0.5	97
3	Stable/mixed	~0.0	~−0.5	+0.8	~0.0	158
4	Late up 2	~−1.5	~−1.0	~0.0	+0.6	71
5	Sustained down 1	~+0.5	~+0.3	~−0.5	~−0.8	91
6	Sustained down 2	~+1.5	~+0.1	~−0.3	~−0.5	133

**Fig 8:**
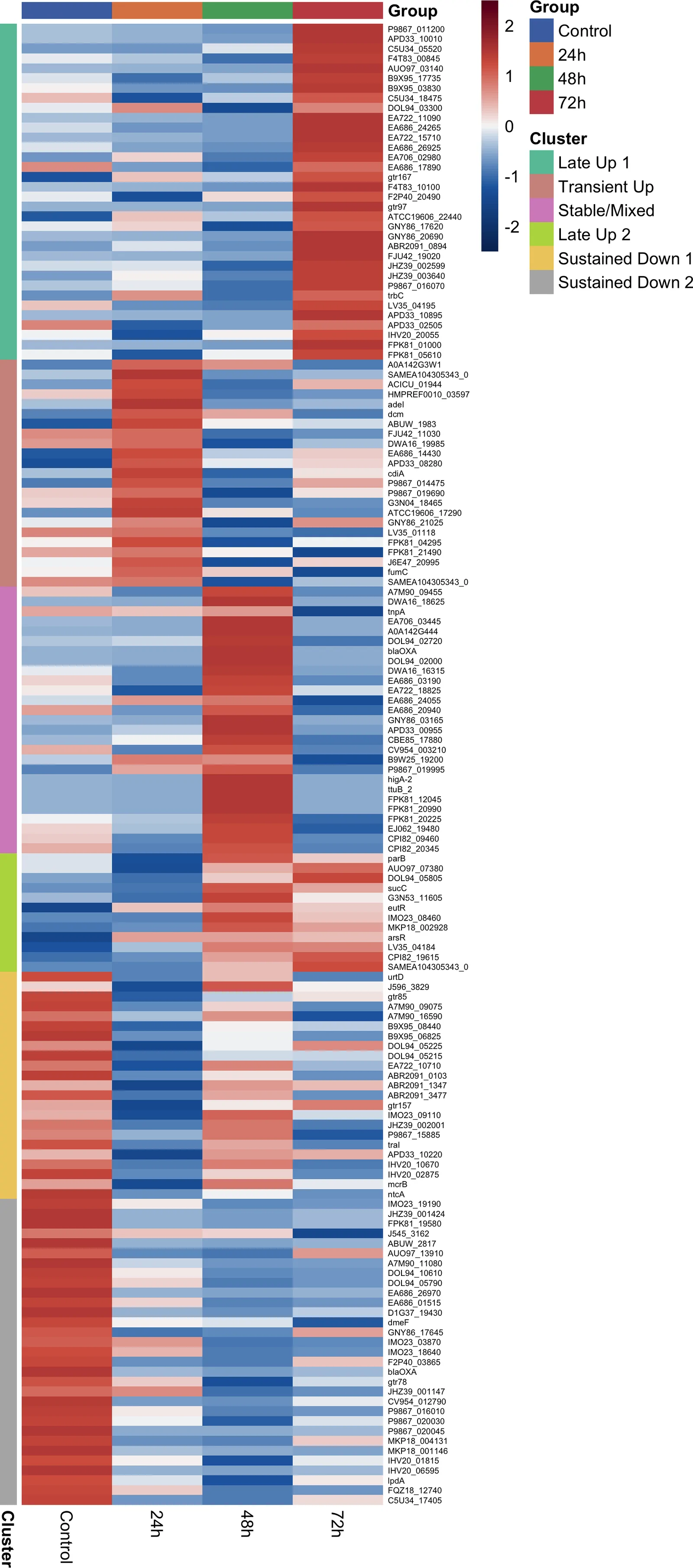
Heatmap of the top 150 highest-variance proteins sorted by cluster assignment. Rows = proteins; columns = four group mean *Z*-score values. Color scale = row-wise *Z*-score (red = high and blue = low). The left-side color bar indicates cluster membership corresponding to Fig. 7.

The transient up cluster (*n* = 97) captured proteins transiently induced at 24 h before declining at subsequent time points, potentially representing immediate early stress response proteins (Fig. 7). The stable/mixed cluster (*n* = 158) exhibited peak expression at 48 h with a return toward baseline at 72 h. The two sustained down clusters (combined *n* = 224) encompassed proteins with comparatively elevated baseline expression in the control condition that declined progressively over the course of CPF4 treatment, suggesting suppression of constitutive metabolic and structural protein functions. A heatmap of the top 150 highest-variance proteins, sorted by cluster assignment (Fig. 8), illustrates the coherence of these temporal trajectory patterns, with cluster boundaries clearly demarcated by an annotation bar on the left.

### Co-expression network analysis

**Fig 9:**
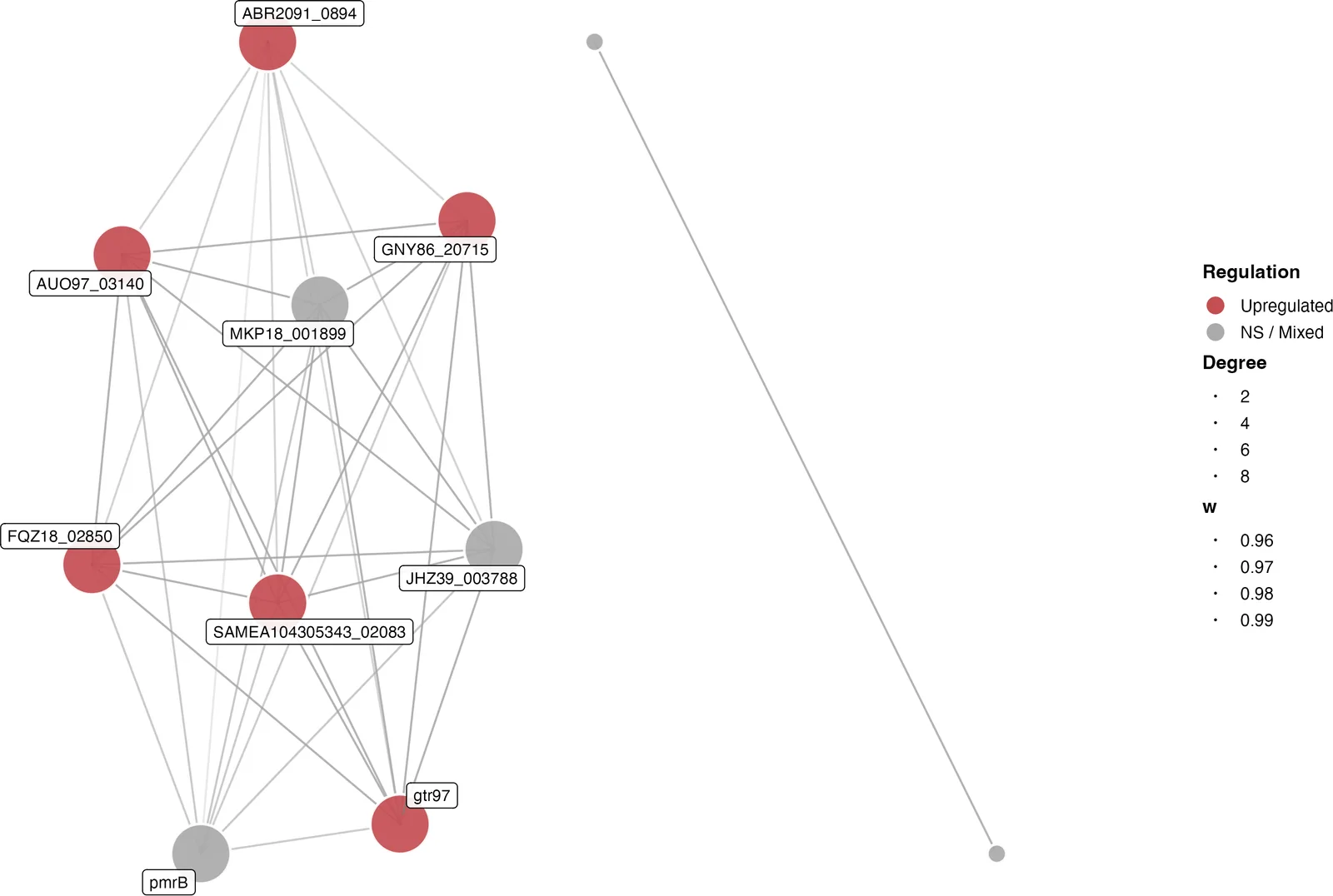
Co-expression network of significant DEPs (union across all comparisons). Nodes = individual proteins; edges connect pairs with Pearson *r* ≥ 0.85 across group mean expression values (11 nodes and 37 edges). Node size = degree centrality. Node color: red = upregulated, blue = downregulated, and gray = NS/mixed at the 72 h vs Control comparison. High-degree hub proteins are labeled.

Co-expression network analysis of all significant DEPs (*n* = 11 unique proteins) using Pearson *r* ≥ 0.85 yielded a network comprising 11 nodes and 37 edges (Fig. 9), indicative of highly coordinated co-expression among the identified DEPs. The network was dominated by a densely connected core module of upregulated proteins, including the HTH domain-containing protein, glycosyltransferase Gtr97, 3-methyladenine DNA glycosylase, DNA helicase, and two uncharacterized proteins (SAMEA104305343_02083 and FQZ18_02850), all of which showed synchronous marked induction at 72 h. Hub proteins with the highest degree centrality—the HTH domain-containing protein and uncharacterized protein SAMEA104305343_02083—were co-expressed with nearly all other network members, supporting their role as central nodes in the 72 h adaptive response. Two peripheral nodes, putative membrane protein ABR2091_0715 and HNH endonuclease LV35_04261, were connected to the main module by a single edge, consistent with their significant downregulation in the 48 h and 72 h comparison.

### Virulence and resistance cross-referencing

**Fig 10:**
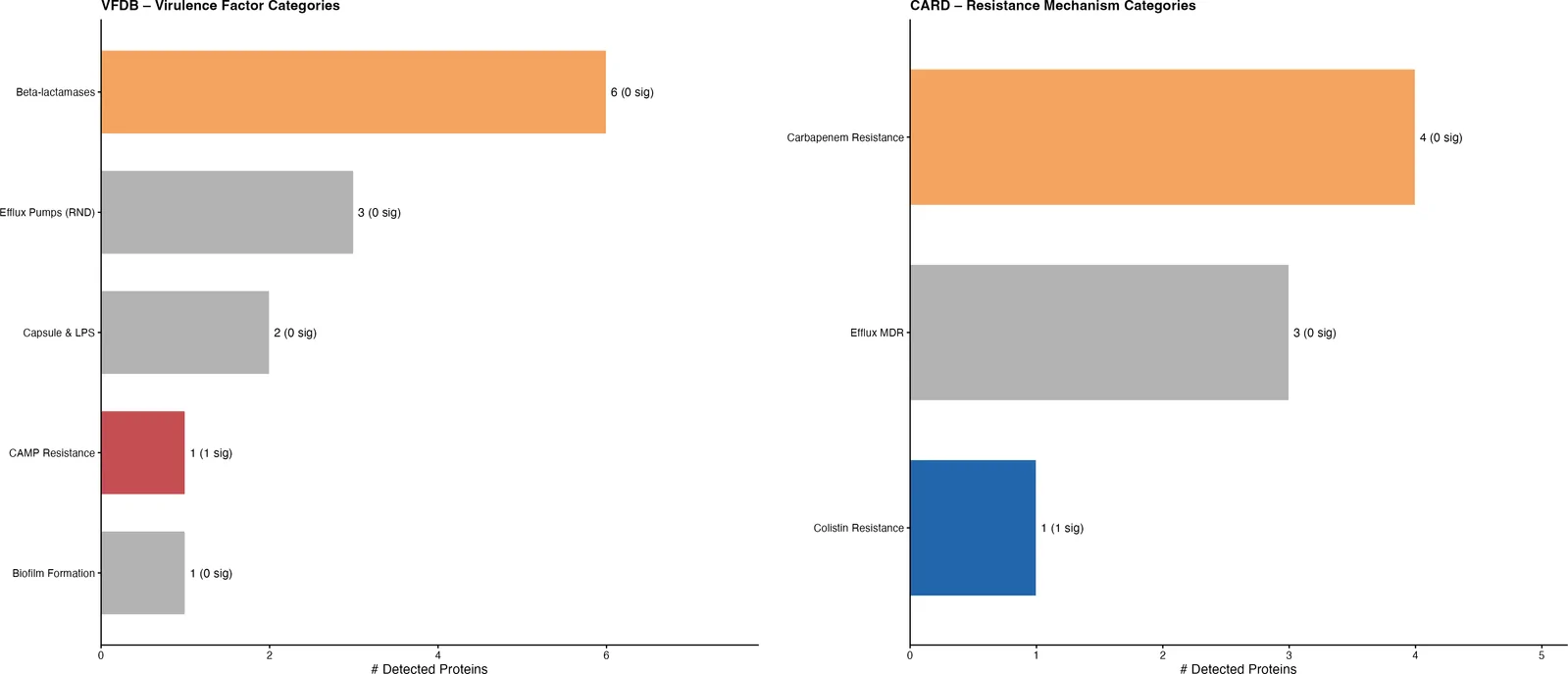
Coverage of virulence factor (VFDB; left) and antibiotic resistance mechanism (CARD; right) categories among all 715 detected proteins. Bar length = number of proteins matching each category. Red/blue bars = category contains ≥ 1 FDR-significant DEP; orange = category contains ≥ 1 nominally significant (*P* < 0.05) but non-FDR-significant protein; gray = detected but not significant.

Cross-referencing all 715 detected proteins against curated VFDB and CARD gene lists revealed five virulence factor categories and three resistance mechanism categories with at least one detected protein (Fig. 10). Notably, the beta-lactamases category (VFDB) contained six detected proteins, and the Carbapenem Resistance category (CARD) contained four detected proteins; both were flagged in orange, indicating that at least one protein per category showed nominal significance (DEqMS *P* < 0.05) without surviving BH-FDR correction. These nominally significant findings should be interpreted with caution, as the limited number of DEPs reduces the statistical power of FDR-controlled analysis.

**Fig 11:**
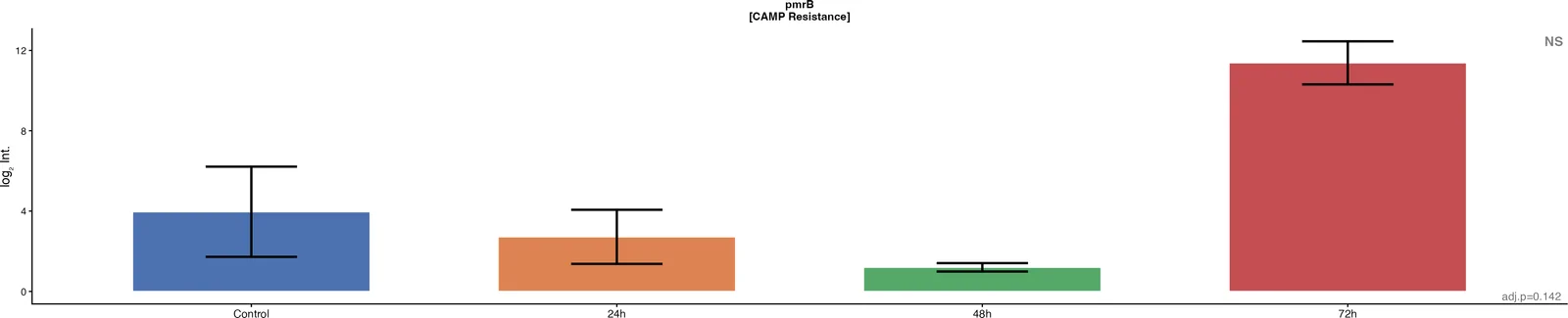
Expression profile of PmrB, the sole FDR-significant DEP matching a virulence/resistance category (CAMP Resistance/Colistin Resistance). Mean log_2_ intensity ± SEM per group. Text in upper right = regulation direction and DEqMS adjusted p-value at 72 h vs Control.

The cationic antimicrobial peptide (CAMP) resistance category (VFDB) and the colistin resistance category (CARD) each contained one FDR-significant DEP (indicated by red and blue bars, respectively): histidine kinase PmrB (log_2_FC = +10.18, adjusted *P* = 0.029 at 72 h vs 48 h), a sensor kinase of the PmrA/PmrB two-component system that regulates lipopolysaccharide modification and mediates colistin resistance in gram-negative bacteria. The expression profile of PmrB (Fig. 11) revealed comparatively elevated expression in the control cluster, progressive suppression during the 24–48 h window, and marked re-induction at 72 h. At the 72 h vs Control comparison, PmrB did not reach FDR significance (adjusted *P* = 0.142), likely due to high inter-replicate variance in the control group. Collectively, these findings suggest that the PmrA/PmrB two-component system contributes to the adaptive resistance response of MDR *A. baumannii* to CPF4 exposure.

## DISCUSSION

This study presents the first quantitative proteomic characterization of the response of MDR *A. baumannii* to CPF4, providing a temporally resolved molecular portrait of how this clinically important nosocomial pathogen adapts to plant-derived antimicrobial challenge. Employing DEqMS, a peptide-count-weighted empirical Bayes framework specifically designed for LFQ LC-MS/MS data with small sample sizes, we identified 25 unique significant DEPs across six pairwise comparisons and uncovered several biologically and clinically relevant adaptive responses.

### Chemical composition and bioactive constituents

Several of the tentatively annotated metabolites detected in CPF4, including lipid-like compounds (fatty acid amide oleamide; C_18_H_35_NO), diterpenoid-related molecules (diterpene kahweol; C_20_H_26_O_3_), fatty acid derivatives (DHA; C_22_H_32_O_2_), phenolic glycosides (glycoside myricitrin; C_21_H_20_O_12_), pyridinium alkaloid (trigonelline; C_7_H_7_NO_2_), and dicarboxylic acid (azelaic acid; C_9_H_16_O_4_), have previously been associated with antimicrobial or membrane-active properties in plant-derived extracts (14). Lipid-related and phenolic compounds have been reported to play important roles in modulating antibiotic resistance in gram-negative bacteria, in part by interfering with lipopolysaccharide modification (15). Lipophilic natural products and terpenoid derivatives are well-established sources of antimicrobial agents due to their capacity to penetrate bacterial membranes, disrupt lipid bilayers, and inhibit key metabolic processes (16). Fatty acids can compromise the structural integrity of the bacterial cell membrane, leading to enhanced membrane permeability and leakage of intracellular constituents, and eventually causing cellular lysis (17). Natural phenolic compounds exert antimicrobial activity through multiple mechanisms, including disruption of microbial cell integrity, impairment of membrane function, and interference with essential metabolic pathways required for bacterial growth and survival (18). The presentation of a hydroxyl group attached to an aromatic ring can contribute to bacterial membrane destabilization, eliciting responses that culminate in biocidal effects (19). However, the untargeted metabolomic approach used in this study does not allow definitive assignment of biological activity to any individual compound. It is therefore more likely that the antibacterial effects of CPF4 arise from the combined activity of multiple co-occurring metabolites rather than from a single dominant constituent. Similar observations have been reported for other complex botanical extracts, where synergistic interactions between chemically diverse compounds contribute substantially to the overall antimicrobial effect (20, 21)

The metabolite profile suggests that CPF4 is enriched in amphiphilic and phenolic-like constituents that may collectively contribute to membrane perturbation and stress induction in gram-negative bacteria. Nevertheless, the specific compounds responsible for the antibacterial activity observed in this study remain unclear and will require further purification and targeted bioactivity-guided investigation. Many amphiphilic lipids and phenolic compounds as identified in CPF4—including oleamide, kahweol, DHA, and myricitrin—are known to affect gram-negative bacteria through membrane destabilization, altered permeability, oxidative stress induction, and disruption of energy metabolism, consistent with the potent antibacterial activity observed against the susceptible *A. baumannii* strain (22). These findings support a multi-target mechanism of antimicrobial action for CPF4 (17, 19). Limitations of the metabolomic analysis should also be acknowledged. Compound annotation in this study was based on untargeted UPLC-MS feature matching without confirmation using authentic standards. Consequently, the reported metabolite identities should be interpreted as tentative. In addition, the detection of several potential environmental or analytical contaminants highlights the need for caution when interpreting low-specificity features. Future studies incorporating targeted metabolomics, compound isolation, and bioactivity-guided fractionation will be important for confirming the chemical composition of CPF4 and identifying the metabolites primarily responsible for its antibacterial activity.

Interestingly, the proteomic responses observed here—particularly the induction of proteins associated with DNA repair and possible membrane remodelling—are broadly consistent with these types of stress responses. Even so, further work involving purified compounds, targeted metabolomics, and functional validation will be necessary to determine which CPF4 constituents are directly responsible for these effects.

### Antimicrobial activity and strain-specific responses

CPF4 exhibited potent bactericidal activity against *A. baumannii* ATCC19606 (MIC/MBC = 31.25 µg/mL) but was ineffective against the MDR clinical isolate (MIC/MBC > 64,000 µg/mL), representing a >2,000-fold difference in susceptibility between the two strains. The MIC value against *A. baumannii* ATCC19606 (31.25 µg/mL) is notably lower than MIC values reported for other *C. pulcherrima* extracts in the literature, which typically range from 2,500 to 15,000 µg/mL for crude extracts (23). This may reflect the enrichment of particularly potent bioactive constituents—such as oleamide, kahweol, and DHA—through fractionation, although the removal of unknown synergistic compounds during this process may simultaneously account for the discrepancy between the MIC observed here and values reported for the crude extracts. This phenomenon has been observed in other plant extract systems. For example, fractionation of *Clitoria ternatea* flower extracts has been shown to reduce antimicrobial potency relative to the crude extract, suggesting that the broader chemical diversity of crude extracts—including constituents that may act synergistically—contributes to their enhanced antimicrobial effect. Conversely, fractionation reduces the number of potentially interfering compounds but may simultaneously separate or remove synergistic bioactive components, resulting in reduced antimicrobial potency and consequently elevated MIC values in fractionated extracts (24).

### Late-phase proteomic response and absence of early DEPs

A notable feature of the data is the complete absence of significant DEPs at both 24 h and 48 h compared to the control under the applied DEqMS significance thresholds. This does not imply absence of biological effect—the clear PCA separation between control and treated groups at both time points (Fig. 2) and the detection of seven DEPs in the 48 h vs 24 h comparison confirm that proteomic changes were occurring. Rather, the absence of significant DEPs vs control at early time points likely reflects the high within-group variability inherent in experiments with *n* = 3 replicates and the conservative nature of DEqMS-based inference under such conditions. The emergence of a robust proteomic response at 72 h, characterized by six highly significant DEPs (adjusted *P* as low as 0.003), indicates that MDR *A. baumannii* mounts a reproducible late-phase adaptive response to sustained CPF4 exposure. This temporal pattern is consistent with a model in which initial bacteriostatic effects suppress overall protein synthesis, followed by the selective induction of adaptive proteins as the bacteria attempt to counter prolonged CPF4-mediated stress (25). MDR *A. baumannii* and related nosocomial pathogens have been shown to shift from acute stress response to chronic persistence strategies through the remodeling of key metabolic pathways, including toxin/antitoxin systems, second messengers, the SOS response, the phenylacetic catabolic pathway, and membrane modification mechanisms (26, 27). Furthermore, as a clinical isolate was employed in this study, the observed proteomic responses at 72 h may also reflect the high adaptive capacity and viability retention characteristic of MDR clinical strains following prolonged exposure to natural antimicrobial compounds or nutrient-limiting conditions (28).

### DNA damage response as a central 72 h adaptation

The convergence of multiple independent lines of evidence implicating the DNA damage response represents the most mechanistically compelling finding of this study. Three of the six significant DEPs identified at 72 h vs Control are functionally linked to DNA repair or nucleic acid processing: (i) 3-methyladenine DNA glycosylase (AUO97_03140), the key initiating enzyme of the base-excision repair pathway; (ii) DNA helicase (ABR2091_0894), which unwinds double-stranded DNA to facilitate repair synthesis; and (iii) helix-turn-helix domain-containing protein (GNY86_20715), which likely regulates the transcriptional induction of the DNA damage response. These findings are corroborated by the FDR-significant enrichment of base-excision repair in GO:BP and alkylbase DNA N-glycosylase activity in GO:MF (Fig. 5).

This DNA damage response is mechanistically consistent with the known phytochemistry of *C. pulcherrima*. The plant is rich in polyphenols and quinones, several of which are known to generate reactive oxygen species (ROS) and reactive electrophiles that cause oxidative DNA base modifications—particularly methylated and oxidized purines, which are canonical substrates for 3-methyladenine DNA glycosylase (29). The coordinated induction of BER machinery at 72 h suggests that CPF4-induced DNA damage has reached a threshold requiring active repair to maintain genome integrity. This response may, however, represent a double-edged adaptation: while BER confers immediate protection, sustained ROS-induced mutagenesis may simultaneously accelerate the evolution of antimicrobial resistance (30).

### Cell surface glycosylation remodeling

The concurrent induction of two glycosyltransferases—Gtr97 (log_2_FC = +9.73) and Gtr162 (log_2_FC = +9.50 at 72 h vs 48 h)—alongside the FDR-significant enrichment of “hexosyltransferase activity” in GO:MF, suggests active remodeling of the bacterial cell surface in response to CPF4 exposure. Glycosyltransferases in gram-negative bacteria are involved in LPS O-antigen biosynthesis and modification, capsular polysaccharide assembly, and protein glycosylation. Modifications to LPS structure represent a well-characterized mechanism of resistance to membrane-active antimicrobials, including certain plant-derived compounds (31). The coordinated induction of cell surface glycosylation enzymes at 72 h, therefore, likely represents a structural defense mechanism, potentially aimed at reducing membrane permeability to hydrophobic extract components or altering the surface charge to repel cationic antimicrobial compounds.

The most significantly upregulated protein, GNY86_20715 (log_2_FC = +10.55), carries a helix-turn-helix DNA-binding domain characteristic of transcriptional regulators in bacteria (32). Its marked FDR-significant induction as the top DEP at 72 h, together with its co-expression with glycosyltransferases, the DNA helicase, and uncharacterized proteins in the co-expression network hub (Fig. 9), suggests that GNY86_20715 may function as a master regulator of the late-phase adaptive response. Characterization of the GNY86_20715 regulon by ChIP-seq or transcriptomic approaches should be prioritized in future mechanistic studies.

Two findings associated with antimicrobial resistance raise important clinical implications. The sensor histidine kinase PmrB was significantly upregulated at 72 h vs 48 h (adjusted *P* = 0.029). The PmrA/PmrB two-component system regulates LPS modification, including the addition of phosphoethanolamine to lipid A, which reduces the binding affinity of colistin and other CAMPs (33). Despite considerable research attention, the mechanisms responsible for colistin resistance in *A. baumannii* remain only partially elucidated. Briefly, colistin is a cationic antimicrobial peptide that binds electrostatically to the negatively charged phosphate groups of lipid A in the outer membrane of gram-negative bacteria (34), displacing stabilizing divalent cations—particularly calcium (Ca^2+^) and magnesium (Mg^2+^)—and disrupting LPS integrity (35). The hydrophobic acyl tail of colistin subsequently inserts into the outer membrane, increasing permeability and enabling further penetration of the inner membrane phospholipid bilayer, ultimately resulting in membrane destabilization and bacterial cell death (36). Induction of PmrB under CPF4-mediated membrane stress may therefore represent an early signal of colistin tolerance acquisition, a finding of particular clinical relevance in the context of extensively drug-resistant (XDR) *A. baumannii* infections. Notably, the natural lignan magnolol has been shown to disrupt the PmrA/B system by dissociating the PmrA regulator from its DNA, thereby preventing the PmrA/B-dependent LPS modification and restoring colistin sensitivity (37). Consistent with this, the expression profile of PmrB in the present study revealed modest suppression during the first 48 h of CPF4 treatment, followed by marked upregulation at 72 h in surviving MDR *A. baumannii* cells, suggesting that PmrB-mediated resistance induction is a last-phase survival response rather than an immediate stress reaction.

### Conclusion

This study presents the first isolation of a *C. pulcherrima*-derived fraction (CPF4) with demonstrated antibacterial activity against MDR *A. baumannii* and provides the first DEqMS-based quantitative proteomic characterization of the bacterial adaptive response to this plant-derived antimicrobial challenge. Metabolomic profiling identified oleamide, kahweol, DHA, myricitrin, trigonelline, and azelaic acid as the predominant bioactive constituents, collectively consistent with a multi-target mechanism of membrane disruption and metabolic interference. Proteomic analysis revealed a temporally structured adaptive response in which significant proteomic remodeling was absent at early time points but emerged robustly at 72 h, characterized by the coordinated induction of DNA damage repair, transcriptional regulation, and cell surface glycosylation machinery. Cross-referencing with resistance databases further implicated the PmrA/PmrB two-component system in late-phase colistin tolerance acquisition under sustained CPF4 exposure, raising important clinical concerns for the management of XDR *A. baumannii* infections. Collectively, these findings advance understanding of the molecular survival strategies employed by MDR *A. baumannii* in response to plant-derived antimicrobial challenge and identify the DNA damage response machinery, cell surface glycosylation enzymes, and the PmrA/PmrB regulatory system as priority targets for further mechanistic and therapeutic investigation. Future studies employing transcriptomic, genetic, and *in vivo* approaches will be essential to validate these proteomic findings and to explore the therapeutic potential of CPF4 and its purified constituents against clinically relevant MDR pathogens.

## Data Availability

The MS/MS raw data and analysis are available in the ProteomeXchange Consortium via the jPOST partner repository: JPST004649 and PXD078836.
